# Quantitative Analysis of Enhanced Computed Tomography in Differentiating Cystitis Glandularis and Bladder Cancer

**DOI:** 10.1155/2020/4930621

**Published:** 2020-06-17

**Authors:** Hui Hua, Yuanxiang Gao, Jizheng Lin, Feng Hou, Jun wei Wang, Yong Zhang, Xuecheng Yang, Hexiang Wang

**Affiliations:** ^1^Department of Thyroid Surgery, The Affiliated Hospital of Qingdao University, Qingdao 266003, China; ^2^Department of Radiology, The Affiliated Hospital of Qingdao University Qingdao, Shandong, China; ^3^Department of Pathology, The Affiliated Hospital of Qingdao University Qingdao, Shandong, China; ^4^Department of Radiology, The Xixiu District People's Hospital of Anshun City, Guizhou, China; ^5^Department of Urology, The Affiliated Hospital of Qingdao University, Qingdao 266003, China

## Abstract

**Objective:**

This study was performed to assess the value of quantitative analysis of enhanced computed tomography (CT) values in the differential diagnosis of bladder cancer and cystitis glandularis (CG).

**Methods:**

Eighty patients with bladder masses (39 with CG and 41 with bladder cancer) who underwent enhanced CT were retrospectively reviewed. The CT enhancement values of the lesion and normal bladder wall in the arterial phase, venous phase, and delayed phase were measured. The relative enhancement CT values (relative enhancement CT value = enhancement CT value of lesion − enhancement CT value of normal bladder) in the arterial phase, venous phase, and delayed phase were also calculated. The pathological results were used as the gold standard, and the area under the curve (AUC), sensitivity, and specificity were calculated for the six groups of quantitative indicators (enhanced CT values and relative enhanced CT values of CG and bladder cancer in the arterial, venous, and delayed phases). We performed the leave-group-out cross-validation method to validate the accuracy, AUC, sensitivity, and specificity. The differences in accuracy, AUC, sensitivity, and specificity among the six groups of quantitative indicators were compared by the *t*-test.

**Results:**

In a combined analysis of the AUC, sensitivity, and specificity performance, the best indicator was the arterial-phase relative enhancement CT value with a cut-off of 25.85 HU (AUC, 0.966; sensitivity, 95.1%; specificity, 92.3%). We used the 100-times leave-group-out cross-validation method to validate the accuracy, AUC, sensitivity, and specificity. Arterial-phase relative enhancement CT values showed the highest AUC and accuracy among the six groups, with statistical significance (*P* < 0.05).

**Conclusion:**

Quantitative analysis of enhanced CT is of great clinical value in the differential diagnosis of CG and bladder cancer.

## 1. Background

Cystitis glandularis (CG) is a proliferative and metaplastic disorder of the bladder mucosa with an incidence of 0.1% to 1.9% in the general population [1]. Bladder cancer is the most commonly occurring urinary tract tumor [2, 3]. On cystoscopic examination, CG often presents as an irregular nodular lesion that sometimes simulates a neoplasm [4]. It is usually misdiagnosed as bladder cancer because its clinical and imaging features mimic those of urothelial carcinoma [5, 6]. Biopsy performed by cystoscopy enables acquisition of only part of the lesion and is therefore inaccurate [5]. The purpose of this study was to evaluate the efficacy of quantitative analysis of enhanced computed tomography (CT) values for differentiating CG from bladder cancer.

## 2. Methods

### 2.1. Patients

The examination protocol was approved by the institutional medical ethics committee, and informed consent was obtained from all patients. Eighty patients with pathologically proven CG and bladder cancer from July 2003 to January 2017 were retrospectively analyzed. The inclusion criteria were performance of an enhanced CT scan, confirmation of the definitive diagnosis by postoperative pathology, and no performance of catheterization. The exclusion criteria were other malignant tumors or serious diseases, a history of urological surgery, poor-quality enhanced CT images, and a lesion area of <20 mm^2^.

In total, 39 patients with CG (34 men and 5 women) were included in the study. Their mean age was 56 ± 14 years (range, 27–75 years). Additionally, 41 patients with bladder cancer (34 men and 7 women) were included. Their mean age was 65 ± 15 years (range, 37–84 years).

### 2.2. Imaging Technology

CT scans were performed using a standard CT protocol for the pelvis. Eighty patients underwent CT scanning after intravenous injection of 80 to 100 ml iopromide 300. CT images were acquired in the arterial phase (30 s after injection), venous phase (50–80 s after injection), and delayed phase (5–30 min after injection). The acquisition parameters were a tube voltage of 120 to 140 kV and tube current of 180 to 200 mAs. All 80 patients underwent cystoscopy or a bladder operation to treat the bladder masses within 1 week after the imaging examination.

### 2.3. Imaging Analysis

Two radiologists who had more than 6 years of experience and who had not been informed of the clinical and histopathological findings independently reviewed the images in consensus. The imaging characteristics were analyzed with respect to location (trigone of the bladder, other parts of the bladder), shape (lobulate or papillary lesions, mound-like lesions), size, margins (well-defined or ill-defined), and attenuation. Signs of extramural infiltration, calcification, necrosis or cyst degeneration, and regional lymph node metastasis were classified as present or absent. Attenuation in the tumors and bladder wall was measured in Hounsfield units (HU) on contrast CT. For evaluation of size, the greatest single dimension of each lesion was measured.

Attenuation in the lesions and bladder wall on CT images was measured. For evaluation of the lesions, >20 mm^2^ oval regions of interest were manually drawn by one radiologist on the image to encompass most of the lesion. We detected the CT value of the lesion with avoidance of blood vessels, artifacts, tumor stalks, necrosis, and margins. Each lesion was measured three times in different areas, and the average CT value was used for the statistical analysis to avoid measurement errors. The relative enhancement CT value was calculated as the enhancement CT value of the lesion minus the enhancement CT value of the normal bladder.

### 2.4. Statistical Analysis

The data were statistically analyzed with SPSS version 17.0 (SPSS Inc., Chicago, IL, USA). Categorical data were analyzed with the *χ*^2^ test. Continuous data are presented as mean and standard deviation and were analyzed by Student's *t*-test. Receiver operating characteristic (ROC) curve analysis was performed to evaluate the diagnostic accuracy of the arterial-, venous-, and delayed-phase enhancement CT values and relative enhancement CT values in the lesion for the differential diagnosis of CG and bladder cancer. ROC curves were used to determine the sensitivity and specificity and extrapolate the optimum cut-off value for CG versus bladder cancer. The differences in the area under the curve (AUC) of the six groups of quantitative indicators (the enhanced CT values and relative enhanced CT values of CG and bladder cancer in the arterial phase, venous phase, and delayed phase) were compared by the *Z* test. A two-tailed *P* value of <0.05 was considered statistically significant. We used open-source software (R v.3.3.1, https://www.r-project) to perform the leave-group-out cross-validation (LGOCV) method to validate the accuracy (ACC), AUC, sensitivity, and specificity. The differences in ACC, AUC, sensitivity, and specificity of the six groups of quantitative indicators (the enhanced CT values and relative enhanced CT values of CG and bladder cancer in the arterial phase, venous phase, and delayed phase) were compared by Student's *t*-test.

## 3. Results

The patient-related and morphologic enhanced CT characteristics of CG and bladder cancer among the 80 patients are listed in Table 1. No differences were found in sex, tumor size, location, calcification, or necrosis or cyst degeneration between CG and bladder cancer (*P* = 0.284–0.945). However, statistically significant differences were found in age, shape, margins, regional lymph node metastasis, and extramural infiltration between CG and bladder cancer (*P* = <0.001–0.047).

**Figure 1:**
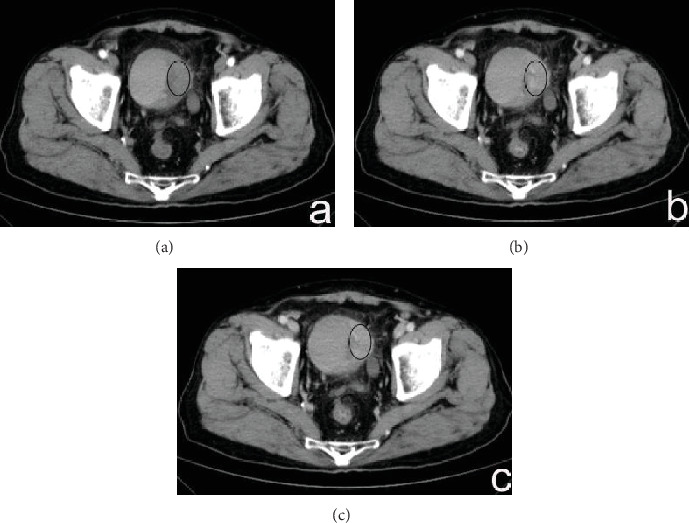
(a–c) A 37-year-old man had CG that presented as mound-like lesions in the left lateral wall of the bladder, including the trigone. The enhanced CT values of the lesions in the arterial, venous, and delayed phases were 43.3, 57.8, and 49.8 HU, respectively. The enhanced CT values in the bladder wall in these three phases were 30.2, 38.9, and 32.5 HU, respectively. The relative enhanced CT values (calculated as enhancement CT values of the lesion − enhancement CT values of normal bladder) in the arterial, venous, and delayed phases were 13.1, 18.9, and 17.3 HU, respectively.

**Figure 2:**
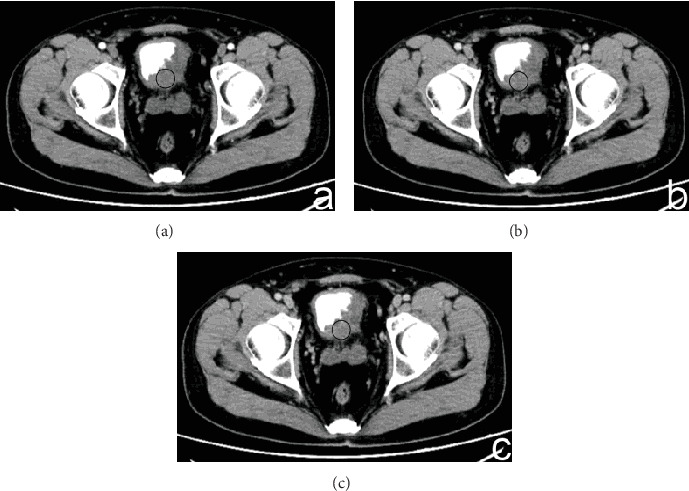
(a–c) A 56-year-old man had bladder cancer that presented as mound-like lesions in the left lateral wall of the bladder, including the trigone. The enhanced CT values of the lesions in the arterial, venous, and delayed phases were 77.2, 83.4, and 79.7 HU, respectively. The enhanced CT values of the bladder wall in these three phases were 31.4, 39.2, and 34.5 HU. The relative enhanced CT values (calculated as enhancement CT values of the lesion − enhancement CT values of normal bladder) in the arterial, venous, and delayed phases were 45.8, 44.2, and 45.2 HU, respectively.

**Figure 3:**
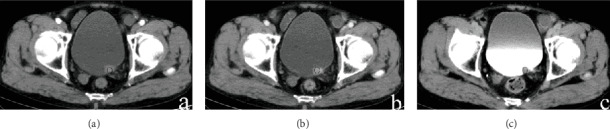
(a–c) A 45-year-old man had bladder cancer that presented as nodule-like lesions in the left trigone. The enhanced CT values of the lesions in the arterial, venous, and delayed phases were 76.2, 81.5, and 76.4 HU, respectively. The enhanced CT values of the bladder wall in these three phases were 32.1, 34.3, and 31.6 HU. The relative enhanced CT values (calculated as enhancement CT values of the lesion − enhancement CT values of normal bladder) in the arterial, venous, and delayed phases were 41.2, 41.5, and 46.4 HU, respectively.

The enhanced CT values of CG (Figure 1) and bladder cancer (Figures 2 and 3) in each phase are listed in Table 2. The differences in the enhanced CT values and relative enhanced CT values in the arterial, venous, and delayed phases were significantly different between CG and bladder cancer lesions (*P* < 0.001).

**Figure 4:**
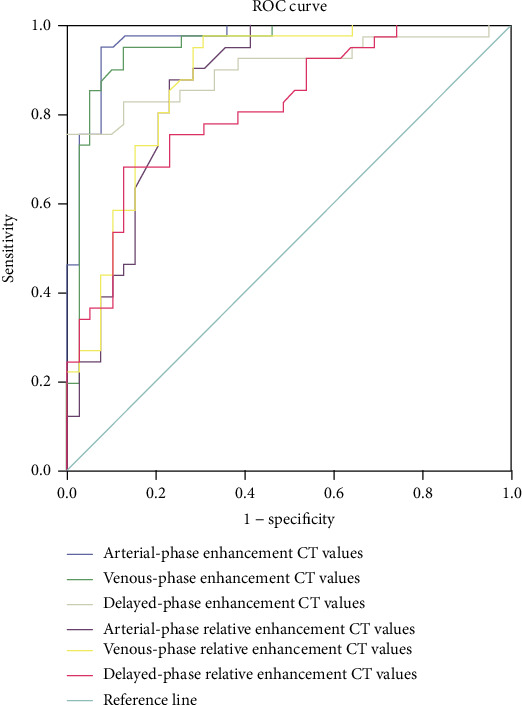
Results of the receiver operating characteristic curve analysis of the six quantitative enhancement CT indicators for differential diagnosis of CG and bladder cancer.

The results of the ROC analysis of the enhancement CT values and relative enhancement CT values in the arterial, venous, and delayed phases for the efficacy of differential diagnosis between CG and bladder cancer are shown in Table 3. The ROC curve is shown in Figure 4, and the AUCs were 0.859, 0.872, 0.812, 0.966, 0.952, and 0.904, respectively. The AUC of the relative enhancement values in the arterial phase was the largest, but there was no difference in the AUC between the relative enhancement values in the other two phases (*Z* = 0.550–1.934, *P* = 0.0531–0.5820). The differential diagnostic efficacy of the relative enhancement CT values in the arterial phase was the best indicator in comprehensive consideration of the AUC, specificity, and sensitivity performance (cut-off value, 25.85 HU; sensitivity, 95.1%; specificity, 92.3%).

**Figure 5:**
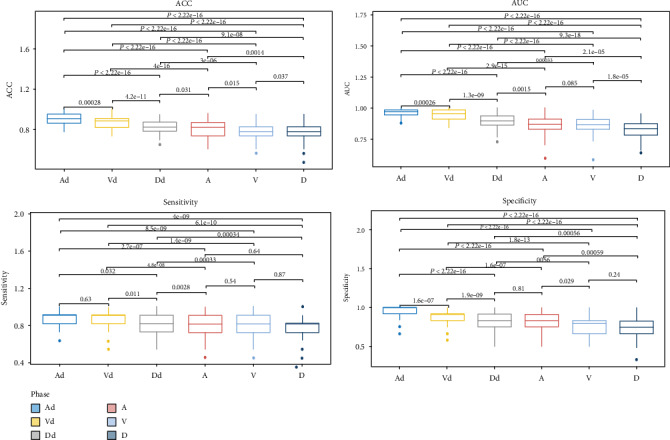
Results of the 100-times LGOCV method for validation of ACC, AUC, sensitivity, and specificity. A: arterial-phase enhancement CT values; V: venous-phase enhancement CT values; D: delayed-phase enhancement CT values; Ad: arterial-phase relative enhancement CT values; Vd: venous-phase relative enhancement CT values; Dd: delayed-phase relative enhancement CT values.

We used the 100-times LGOCV method to validate the ACC, AUC, sensitivity, and specificity, and the results are shown in Table 4. The AUC in the six groups ranged from 0.824 to 0.963, ACC ranged from 0.763 to 0.914, sensitivity ranged from 0.776 to 0.877, and specificity ranged from 0.748 to 0.953. The arterial-phase relative enhancement CT values showed the highest AUC and ACC among the six groups, and the differences were statistically significant (*P* < 0.05). Although the arterial-phase relative enhancement CT values also showed relatively high sensitivity and specificity, the differences among the six groups were not statistically significant (*P* > 0.05) (Figure 5).

## 4. Discussion

The etiology and pathogenesis of CG are unclear. CG is caused by metaplasia of transitional epithelium to glandular epithelium. Additionally, some authors have reported that these masses can progress to bladder adenocarcinoma [6–8]. Patients with CG usually have hematuria as well as CT, ultrasound, and magnetic resonance imaging findings of diffuse bladder wall thickening or nodules, similar to those of bladder malignancy [5, 9]. Transurethral resection is recommended for treatment of CG [10]. However, radical cystectomy and local lymph node dissection are recommended for muscle-invasive bladder cancer, and local cystectomy for bladder preservation with immediate postoperative lavage chemotherapy is recommended for non-muscle-invasive bladder cancer. Radical cystectomy is still required for recurrent postoperative episodes [11–13]. The treatment and prognosis differ between CG and bladder cancer. Therefore, clarifying the diagnosis before surgery is very important.

The results of this study showed no statistically significant differences in sex, location, tumor size, calcification, or necrosis or cyst degeneration between CG and bladder cancer. However, statistically significant differences were found in age, shape, margins, regional lymph node metastasis, and extramural infiltration between CG and bladder cancer. A lobulate or papillary shaped bladder lesion with ill-defined margins in an older patient may be the features that differentiate bladder cancer from CG. However, these features cannot serve as differential diagnostic criteria because in clinical practice, all of these features overlap between the two conditions. Extramural infiltration and regional lymph node metastasis might serve as differential diagnostic criteria because they only occur in bladder cancer. Additionally, according to a previous study, extramural infiltration into an intact muscle layer is a feature that differentiates CG from urothelial carcinoma [14]. However, when bladder cancer encroaches on surrounding structures or is associated with regional lymph node metastasis, the tumor is already in the middle to late stage, which will greatly reduce the significance of diagnosis and treatment of the disease. Therefore, there are no specific features for the differential diagnosis of the two conditions, particularly early-stage bladder cancer versus CG.

This study showed that the degree of enhancement of six groups of quantitative indicators in patients with CG was lower than that in patients with bladder cancer, which may be related to the blood supply of CG and bladder cancer. In contrast to malignant tumors, CG exhibits proliferative changes of the bladder mucosa, such as epithelial crypts, submucosal masses of epithelial cells (von Brunn's nests), and subepithelial fluid-filled cysts associated with cystitis cystica [15]. Although CG is a precancerous lesion, it does not show rapid tumor-like proliferation and is still considered a type of inflammation; thus, its enhancement density is relatively low. Bladder cancer is richly vascular, so its enhancement is more significant [16].

In the present study, an ROC curve analysis was used to perform a quantitative analysis of six groups of data: the enhanced CT values and relative enhanced CT values of CG and bladder cancer in the arterial phase, venous phase, and delayed phase. The AUC of the relative enhanced CT value in the arterial phase was the largest. However, the AUCs of the relative enhanced CT values in the arterial phase, venous phase, and delayed phase were not significantly different. We used the LGOCV method to validate the AUC, ACC, sensitivity, and specificity. The results showed that the relative enhanced CT value in the arterial phase had the best performance and AUC and ACC had statistic difference between six groups. Thus, the relative enhancement value in the arterial phase was the best indicator (cut-off value, 25.85 HU; AUC, 0.966; sensitivity, 95.1%; specificity, 92.3%). This may have been because the degree of arterial enhancement mainly depends on the microvascular density (i.e., degree of vascularization) [17], and the formation and development of bladder cancer with vascular dependence has more than the bladder wall of new blood vessels. The attenuation values of bladder cancer on contrast-enhanced CT at 40 to 45 s after initiation of contrast agent injection may represent vascularity [17]; this vascularity period is according to the arterial phase scanning time in our study. Additionally, it may explain why the differential diagnostic efficacy of the relative enhancement CT value in the arterial phase was superior to the other indicators in comprehensive consideration of the AUC, specificity, and sensitivity performance. Previous studies have also suggested that measurement of the expression of hormone receptors such as estrogen receptor alpha may assist in the diagnosis of primary bladder cancer [18]. However, such measurements are not in widespread use because of susceptibility to differences in experimental procedures and interobserver variability.

This study has some limitations. First, the sample size was small, and the results require further verification by expanding the sample and including multiple centers. Second, the scan time in the delayed phase had a wide range (5–30 min after injection). Finally, this study only focused on the difference in CT values between CG and bladder cancer, and the differential diagnosis between bladder cancer and other benign bladder lesions, such as chronic bladder inflammation or bladder malakoplakia, needs to be further studied.

## 5. Conclusions

Quantitative analysis of enhanced CT values was valuable for the differential diagnosis of CG versus bladder cancer.

## Figures and Tables

**Table 1:** Patient-related and morphologic enhanced CT characteristics of CG and bladder cancer.

Characteristic	CG (*n* = 39)	Urothelial carcinoma	*P*
		(*n* = 41)	
Age (years)	56.46 ± 13.68	65.37 ± 15.27	<0.001
Sex			
Female	5	7	0.284
Male	34	34	
Size (mm)	3.66 ± 1.31	3.76 ± 1.24	0.715
Location			
Trigone of the bladder	20	20	0.823
Other part of the bladder	19	21	
Shape			
Lobulate or papillary lesions	20	31	0.024
Mound-like lesions	19	10	
Margin			
Well-defined	27	17	0.013
Ill-defined	12	24	
Calcification			
+	4	3	0.945
_	35	38	
Necrosis or cyst degeneration			
+	5	7	0.594
-	34	34	
Enlarged lymph node			
+	0	4	0.047
-	39	37	
Extramural infiltration			
+	0	6	0.014
-	39	35	

Data are presented as mean ± standard deviation or number of patients. Age and size were compared using independent *t*-tests. All other parameters were analyzed using *χ*^2^ tests. CT: computed tomography; CG: cystitis glandularis.

**Table 2:** Enhanced CT values of CG and bladder cancer.

Variable	Group		*P* value
	CG	Urothelial carcinoma	
Arterial-phase enhancement CT values	56.02 ± 15.40 HU	76.63 ± 11.94 HU	<0.001
Venous-phase enhancement CT values	61.45 ± 15.28 HU	84.11 ± 13.27 HU	<0.001
Delayed-phase enhancement CT values	54.12 ± 13.36 HU	70.95 ± 13.33 HU	<0.001
Arterial-phase relative enhancement CT values	11.25 ± 9.79 HU	38.89 ± 14.67 HU	<0.001
Venous-phase relative enhancement CT values	15.94 ± 10.34 HU	43.54 ± 15.90 HU	<0.001
Delayed-phase relative enhancement CT values	12.04 ± 7.23 HU	33.66 ± 14.43 HU	<0.001

Data are presented as mean ± standard deviation, and all variables were compared using independent *t*-tests. CT: computed tomography; CG: cystitis glandularis.

**Table 3:** Results of ROC analysis for CG and bladder cancer.

Variable	Sensitivity (%)	Specificity (%)	Cut-off score (HU)	AUC (95% CI)
Arterial-phase enhancement CT values	82.9	76.9	65.95	0.859 (0.763–0.926)
Venous-phase enhancement CT values	82.9	76.9	71.65	0.872 (0.710–0.891)
Delayed-phase enhancement CT values	78	69.2	57.85	0.812 (0.779–0.936)
Arterial-phase relative enhancement CT values	95.1	92.3	25.85	0.966 (0.899–0.994)
Venous-phase relative enhancement CT values	95.1	87.2	26.85	0.952 (0.880–0.987)
Delayed-phase relative enhancement CT values	82.9	76.9	18.35	0.904 (0.817–0.959)

ROC: receiver operating characteristic; CG: cystitis glandularis; AUC: area under the curve; CI: confidence interval; CT: computed tomography.

**Table 4:** Results of the 100-times LGOCV method for validation of ACC, AUC, sensitivity, and specificity.

Variable	AUC	ACC	Sensitivity	Specificity
Arterial-phase enhancement CT values	0.868 ± 0.0737	0.799 ± 0.0769	0.787 ± 0.127	0.809 ± 0.128
Venous-phase enhancement CT values	0.866 ± 0.0665	0.773 ± 0.0733	0.776 ± 0.127	0.769 ± 0.129
Delayed-phase enhancement CT values	0.824 ± 0.067	0.763 ± 0.0783	0.779 ± 0.117	0.748 ± 0.118
Arterial-phase relative enhancement CT values	0.963 ± 0.0293	0.914 ± 0.0475	0.871 ± 0.0915	0.953 ± 0.0684
Venous-phase relative enhancement CT values	0.943 ± 0.0454	0.886 ± 0.0598	0.877 ± 0.0944	0.893 ± 0.0863
Delayed-phase relative enhancement CT values	0.897 ± 0.0554	0.821 ± 0.0699	0.839 ± 0.115	0.805 ± 0.110

Data are presented as mean ± standard deviation. LGOCV: leave-group-out cross-validation; ACC: accuracy; AUC: area under the curve; CT: computed tomography.

## Data Availability

The data used to support the findings of this study are available from the corresponding author upon request.
